# Impact of pre-existing dengue immunity on human antibody and memory B cell responses to Zika

**DOI:** 10.1038/s41467-019-08845-3

**Published:** 2019-02-26

**Authors:** Paulina Andrade, Ciara Gimblet-Ochieng, Faraz Modirian, Matthew Collins, Maritza Cárdenas, Leah C. Katzelnick, Magelda Montoya, Daniela Michlmayr, Guillermina Kuan, Angel Balmaseda, Josefina Coloma, Aravinda M. de Silva, Eva Harris

**Affiliations:** 1 Division of Infectious Diseases and Vaccinology, School of Public Health, University of California, Berkeley, Berkeley, CA 94720-3370 USA; 2 Colegio de Ciencias Biológicas y Ambientales, Universidad San Francisco de Quito, Quito, EC170157 Ecuador; 3 Department of Microbiology and Immunology, University of North Carolina, Chapel Hill, NC 27599-7292 USA; 4 Centro de Salud Sócrates Flores Vivas, Ministry of Health, Managua, 12014 Nicaragua; 5 Sustainable Sciences Institute, Managua, 14007 Nicaragua; 6 Laboratorio Nacional de Virología, Centro Nacional de Diagnóstico y Referencia, Ministry of Health, Managua, 16064 Nicaragua; 7 Present Address: Hope Clinic of the Emory Vaccine Center, Division of Infectious Diseases, Department of Medicine, School of Medicine, Emory University, Decatur, GA 30030 USA

## Abstract

Little is known about enduring memory B cell (MBC) responses to Zika virus (ZIKV) and their relationship with circulating antibodies. Here we comprehensively assess MBC frequency and specificity alongside serum binding and neutralizing antibody responses to ZIKV ~2 weeks and ~8 months postinfection in 31 pediatric subjects with 0, 1 or >1 prior infections with the related dengue virus (DENV). ZIKV infection elicits a robust type-specific MBC response, and the majority of late convalescent anti-ZIKV serum neutralizing activity is attributable to ZIKV-specific antibodies. The number of prior DENV infections does not influence type-specific or cross-reactive MBC responses, although ZIKV has the highest cross-reactivity with DENV3. DENV cross-reactive MBCs expanded by ZIKV infection decline in number and proportion by late convalescence. Finally, ZIKV induces greater cross-reactivity in the MBC pool than in serum antibodies. Our data suggest immunity to DENV only modestly shapes breadth and magnitude of enduring ZIKV antibody responses.

## Abstract (web summary)

Here, Andrade et al. assess the memory B cell (MBC) and antibody response to Zika virus (ZIKV) in individuals with and without prior dengue virus (DENV) infection and find that ZIKV infection elicits a robust and specific MBC response that is only modestly affected by the number of prior DENV infections.

## Introduction

The flaviviruses comprise a genus of arthropod-borne viruses that cause extensive endemic and epidemic human disease worldwide. Dengue virus (DENV) places an estimated 3.9 billion people in 128 countries at risk of infection, and up to ~100 million dengue cases occur annually^1^. The four serotypes of DENV (DENV1–4) cause a spectrum of illness ranging from classic dengue fever to the potentially fatal Dengue Shock Syndrome^2^. Starting in 2014, Zika virus (ZIKV) emerged and spread rapidly to many countries, affecting millions of susceptible individuals, especially in the Americas^3,4^. As DENV and ZIKV share the same ecology and mosquito vectors, the same populations are at risk of infection by these viruses. A minority of ZIKV infections are symptomatic, and these present clinically similarly to other flavivirus infections such as dengue^5^. However, ZIKV can also cause more severe disease, including microcephaly and other congenital birth defects when infection occurs in utero, as well as Guillain–Barré syndrome in adults^6–8^.

The flavivirus positive-sense RNA genome encodes for three structural proteins, capsid (C), premembrane/membrane (prM/M), and envelope (E)—the main target of B cells and protective antibodies—and seven nonstructural proteins^9^. E protein amino acid sequences differ by as much as 40% between DENV serotypes^10^, and by 41–46% from ZIKV^11^. DENV-infected individuals develop B cells targeting epitopes shared between serotypes (cross-reactive) and B cells targeting epitopes unique to each serotype (type-specific)^12,13^. Upon resolution of infection, activated B cells give rise to two distinct and enduring populations of antigen-specific cells: memory B cells (MBCs) and serum antibody-producing long-lived plasma cells (LLPCs)^14^. Mouse studies have demonstrated nonredundant function and distinct breadth of antigen specificity for MBCs and LLPCs, with greater cross-reactivity within the MBC pool and greater type-specificity within the serum antibody population following flavivirus infection^15^. However, the relationship of LLPCs and MBCs remains understudied in humans, particularly in the context of sequential infection by closely related pathogens such as DENV and ZIKV. Evaluating the relative contribution of MBCs and LLPCs to protection and disease has been hindered by a lack of studies systematically characterizing MBCs side-by-side with serum antibody responses.

A hallmark of flavivirus infections is the induction of MBC and serum antibodies that cross-react with other flaviviruses. Protection from secondary dengue is a balance that depends in part on the quality and quantity of pre-existing type-specific and cross-reactive serum antibodies as a first line of defense^16^ and possibly on secreted antibodies from rapidly reactivated MBCs as a second line of defense^17^. Serum responses, especially neutralizing antibodies, have been correlated with protection in flavivirus vaccine development as well as in natural infection studies^16,18–20^. Specific low-to-intermediate levels of cross-reactive antibodies induced by primary DENV infections have been implicated in enhanced viral replication and severe disease during a secondary infection with a new serotype^21,22^. People who have recovered from secondary DENV infections maintain high levels of serotype cross-reactive MBCs and serum antibodies^23^ that are correlated with long-lasting, cross-protective immunity to all four DENV serotypes. Although ZIKV is closely related to the DENV serocomplex, whether lessons learned from DENV about antibody cross-reactivity and pathogenesis also apply to ZIKV is still under investigation. Human antibodies induced by DENV infections that cross-react with ZIKV have been shown to be protective or enhancing in different cell culture and animal models of ZIKV infection^11,24–27^. However, in human and nonhuman primate studies published to date, DENV infection history has not been associated with severity of ZIKV infection and disease^28–31^.

A few studies have described either serum antibody responses or isolated monoclonal antibodies (mAbs) from the MBC pool in ZIKV-infected individuals with and without prior DENV immunity^25,32–35^. Several mAbs have been identified targeting cross-reactive or type-specific epitopes, but these mAbs were from single time-point samples from a small number of individuals without detailed information about DENV infection history. Regarding serum antibodies, ZIKV cross-reactivity is present during primary and secondary dengue with modest cross-neutralization during early convalescence that in some cases is maintained over time^24,26,36,37^. Low levels of cross-reactivity and cross-neutralization to DENV are also present during and after ZIKV-infection, with higher titers observed in people with prior DENV immunity^36,37^. However, the contribution of cross-reactive antibodies to ZIKV neutralization capacity in ZIKV-infected individuals from endemic areas has not yet been defined. Further, the full extent of MBC and serum antibody cross-reactivity between the DENV serocomplex and ZIKV and the influence of pre-existing DENV immunity on the enduring MBC and antibody response to ZIKV infection in individuals in endemic areas remains poorly understood.

In this work, we analyzed MBC frequency and specificity at the single-cell level alongside the serum antibody response to ZIKV infection in a well-characterized cohort of pediatric patients ongoing for 15 years with known prior DENV infection history from a dengue-endemic setting to address fundamental questions about properties of type-specific and cross-reactive serum antibodies and MBCs at a polyclonal level. We show that despite phylogenetic and antigenic relatedness to DENV, ZIKV infection generates a strong type-specific response in the MBC repertoire and neutralizing antibody response in serum that is maintained into late convalescence. We also find that the number of previous DENV exposures does not influence the development of type-specific or cross-reactive responses during ZIKV infection. We demonstrate that a strong ZIKV and DENV cross-reactive response in the MBC population at early convalescence declines in magnitude and proportion by late convalescence, although greater DENV-ZIKV cross-reactivity is maintained in the MBC pool than in serum antibodies. Finally, we find that in this population, ZIKV displays higher cross-reactivity toward DENV3 than to other serotypes at the MBC level.

## Results

### Differential recognition of ZIKV and DENV by ZIKV+ MBCs

We first interrogated MBCs to discern the proportion and magnitude of type-specific and cross-reactive MBCs in patients who acquired reverse transcription polymerase chain reaction (RT-PCR)-confirmed ZIKV infection (ZIKV+) following a history of no (DENV-naïve/ZIKV), one (DENV-ZIKV), or more than one (+DENV-ZIKV) prior DENV infection (Table 1). The previous DENV infection history of the ZIKV+ patients was known because the subjects were enrolled in a long-term prospective dengue study (see Methods).

**Table 1:** Characteristics of ZIKV-infected patients

	DENV-naïve	1 prior DENV infection^a^	>1 prior DENV infection^b^
Number, *n*	11	12	8
Sex, *n* (%) female	3 (27.3)	6 (50)	3 (37.5)
Age in years, mean (SD)	9 (2.55)	11 (1.8)	10 (3.5)
Day of early convalescent sample collection^c^, mean (SD)	15 (6.2)	14 (4.0)	14 (4.4)
Month of late convalescent sample collection^d^, mean (SD)	8 (1.5)	8 (1.6)	7 (2)

^a^Designated DENV-ZIKV group.

^b^Designated +DENV-ZIKV group.

^c^Days postonset of symptoms.

^d^Months postonset of symptoms.

**Fig. 1:**
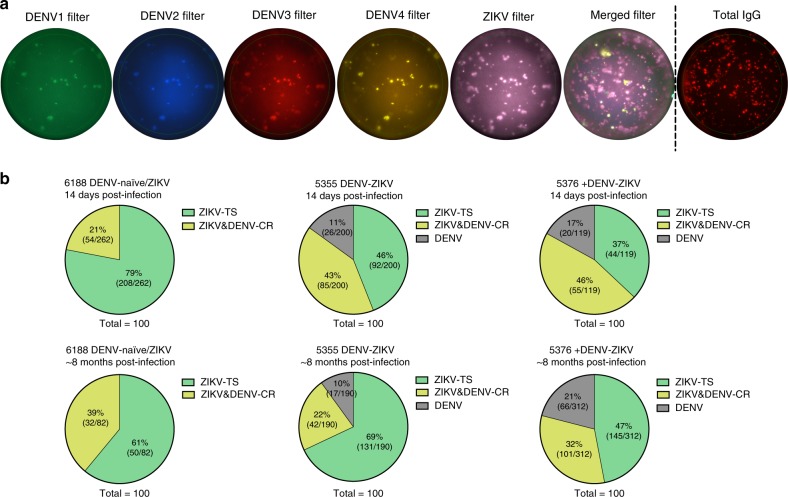
Type-specific and cross-reactive IgG-secreting MBC in ZIKV+ patients. MBC responses in patients with RT-PCR-confirmed ZIKV infection and no (DENV-naïve/ZIKV), one (DENV-ZIKV), or more than one (+DENV-ZIKV) prior DENV infection(s) were analyzed by the Multi-Color FluoroSpot assay at ~14 days and ~8 months postinfection. **a** Representative images of activated MBCs from patient 6188 (DENV-naïve/ZIKV) after ~14 days postinfection. Antigen specificity of MBC responses to ZIKV and the four DENV serotypes are shown as spots represented in each filter. The total ASC of activated MBCs was analyzed in parallel (Total IgG). **b** Proportion of antigen-reactive MBCs to ZIKV, ZIKV and DENV, and DENV in representative patients from the DENV-naïve/ZIKV, DENV-ZIKV, and +DENV-ZIKV groups at ~14 days and ~8 months postinfection. ZIKV-TS, MBCs that react with ZIKV and not with any DENV serotype; ZIKV&DENV-CR, MBCs that react with ZIKV and at least with one DENV serotype; DENV, MBCs that react with one or more DENV serotypes but not with ZIKV

We used a modified Multi-Color FluoroSpot assay to determine the MBC specificity at the single-cell level for all four DENV serotypes and ZIKV^38^. In order to measure the MBC response as it transitions from early to late convalescence, we detected ZIKV- and/or DENV-specific IgG-producing activated MBCs from patients at ~14 days and ~8 months postinfection (Fig. 1a). Total IgG-producing activated MBCs were measured in parallel, denominated activated antibody-secreting cells (ASC) (Fig. 1a, right). Peripheral blood mononuclear cells (PBMCs) from flavivirus-naïve patients were also analyzed by the multi-color FluoroSpot as negative controls (Supplementary Fig. 1 and 2a). In this study, we defined MBCs as cross-reactive if IgG binding was detected in the filter for ZIKV and for at least one DENV serotype, whereas a type-specific MBC clone produced IgG that only bound to ZIKV. A representative individual from each infection group is shown in Fig. 1 and Fig. S3a, and all responses are shown in Supplementary Fig. 3b (DENV-naïve/ZIKV, *n* = 11; DENV-ZIKV, *n* = 12; +DENV-ZIKV, *n* = 8). The Multi-Color FluoroSpot results showed that MBCs were predominantly ZIKV type-specific in the DENV-naïve/ZIKV subject 6188 at ~14 days and ~8 months postinfection (79% and 61%, respectively) (Fig. 1b). Subjects 5355 (DENV-ZIKV) and 5376 (+DENV-ZIKV), who had been previously infected with DENV, displayed ZIKV type-specific responses of 46% and 37%, respectively, at ~14 days postinfection that increased in proportion over time (69% and 47%, respectively). ZIKV-DENV cross-reactive MBCs accounted for a higher fraction of antigen-reactive MBCs from subjects 5355 and 5376 at ~14 days postinfection (43% and 46%, respectively), but not after ~8 months postinfection (22% and 32%, respectively) (Fig. 1b). The DENV-reactive response (i.e., MBC-derived IgG binding to any combination of one or more DENV serotypes, but not reacting to ZIKV) in subjects 5355 and 5376 were 11% and 24%, respectively, at ~14 days postinfection and remained stable or decreased slightly in both patients ~8 months postinfection (9% and 21%, respectively). Similar results were observed in another set of representative patients as well (Supplementary Fig. 3a). Antibodies secreted from MBCs in supernatants from activated PBMCs from representative patients from each group demonstrated reactivity to antigens from all four DENV serotypes and ZIKV, displaying higher titers to ZIKV than to DENV at the binding level and also showing ZIKV neutralizing capacity but mostly undetectable DENV neutralizing antibodies (Supplementary Fig. 2). Our results suggest that in both DENV-naive and DENV-immune individuals, a substantial proportion of the MBC and MBC-derived antibody responses to ZIKV recognize type-specific epitopes in early and late convalescence.

### ZIKV type-specific MBCs are maintained in ZIKV+ patients

**Fig. 2:**
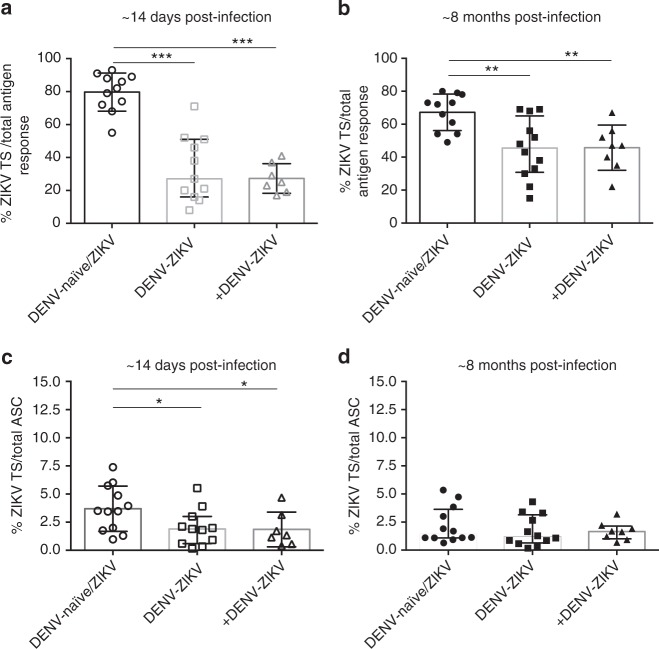
ZIKV type-specific MBC response is maintained over time in ZIKV+ patients. ZIKV type-specific MBC cell responses in ZIKV-infected patients with no (DENV-naïve/ZIKV), one (DENV-ZIKV), or more than one (+DENV-ZIKV) prior DENV infection. **a**, **b** ZIKV type-specific IgG-secreting MBC response over total antigen response at ~14 days (**a**) and ~8 months (**b**) postinfection. **c**, **d** ZIKV type-specific IgG-secreting MBC response over total ASC response at ~14 days (**c**) and ~8 months (**d**) postinfection. The median and interquartile range (IQR) are shown for all graphs. Significance was determined by Kruskal–Wallis test and Dunn’s test; *p < 0.05, **p < 0.01, ***p < 0.001. DENV-naïve/ZIKV, *n* = 11; DENV-ZIKV, *n* = 12; +DENV-ZIKV, *n* = 7 at ~14 days postinfection (one PBMC sample was not viable) and *n* = 8 at ~8 months postinfection

We next analyzed ZIKV type-specific responses in greater detail. We found that ZIKV type-specific MBCs were generated early and were maintained over time in each group of patients regardless of DENV immunity. The DENV-naïve/ZIKV group had a significantly greater proportion of type-specific MBCs out of the flavi-reactive memory pool (78%) compared to the DENV-ZIKV (32%) and +DENV-ZIKV groups (26%) (*p* < 0.001 for both groups, Kruskal–Wallis test and Dunn’s test) ~14 days postinfection (Fig. 2a). At ~8 months postinfection, the same pattern was detected; nonetheless, a substantial proportion (~40–50%) of the MBC response was ZIKV type-specific in both DENV-immune groups (Fig. 2b) with the percent of ZIKV type-specific MBCs in the DENV-naïve/ZIKV, DENV-ZIKV, and +DENV-ZIKV groups as 65%, 37.5% and 47%, respectively (*p* < 0.05, Kruskal–Wallis test and Dunn’s test). DENV-naïve subjects also mounted a significantly higher magnitude ZIKV type-specific MBC response (out of the total ASC memory pool) compared to the DENV-immune groups at ~14 days postinfection (median 3.5%, 1.9%, and 1.3%, *p* < 0.05, Kruskal–Wallis test and Dunn’s test, Fig. 2c); however, this difference dissipated after ~8 months postinfection, and ZIKV type-specific responses were maintained in all groups at a similar level (Fig. 2d). Interestingly, ZIKV type-specific MBC responses did not decrease significantly in magnitude between early and late convalescence in any group (Supplementary Fig. 4).

### ZIKV and DENV cross-reactive MBCs decrease over time

**Fig. 3:**
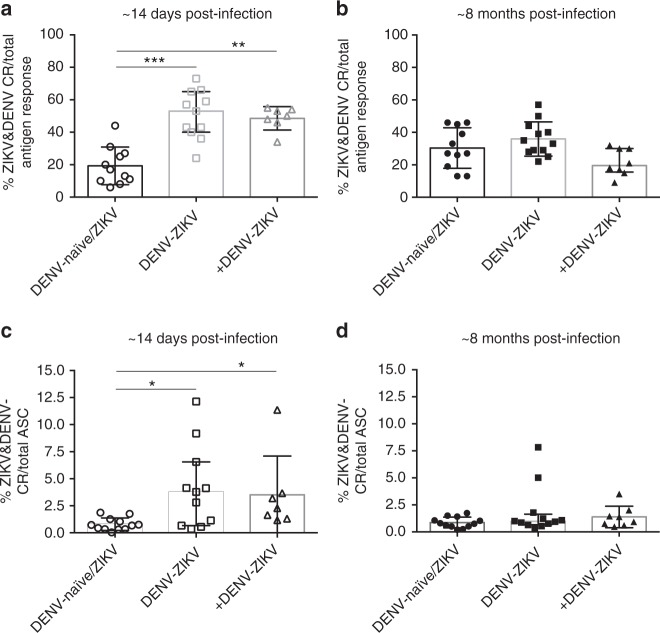
ZIKV and DENV cross-reactive MBCs decrease over time in ZIKV+ patients. ZIKV and DENV cross-reactive B cell responses in ZIKV-infected patients with no (DENV-naïve/ZIKV), one (DENV-ZIKV), or more than one (+DENV-ZIKV) prior DENV infection are shown. **a**, **b** ZIKV and DENV cross-reactive IgG-secreting MBC responses over total antigen response at ~14 days (**a**) and ~8 months (**b**) postinfection. **c**, **d** ZIKV and DENV cross-reactive IgG-secreting MBC response over total ASC response at ~14 days (**c**) and ~8 months (**d**) postinfection. Median and IQR are shown for all graphs. Significance was determined by Kruskal–Wallis test and Dunn’s test; *p < 0.05, **p < 0.01, ***p < 0.001. DENV-naïve, *n* = 11; DENV-ZIKV, *n* = 12; +DENV-ZIKV, *n* = 7 at ~14 days postinfection (one PBMC sample was not viable) and *n* = 8 at ~8 months postinfection

We next asked whether ZIKV infection stimulated cross-reactive MBCs from previous DENV infections and expanded the size of the cross-reactive MBC pool. At early convalescence (~14 days post-ZIKV infection), ZIKV and DENV cross-reactive MBCs constitute 40–50% of the total flavi-reactive MBCs in people with one or more previous DENV infection. This proportion was significantly higher than the 20% of cross-reactive MBCs present in DENV-naïve/ZIKV (*p* < 0.001 and *p* < 0.01, respectively; Kruskal–Wallis test and Dunn’s test, Fig. 3a). However, over time, the percentage of cross-reactive MBCs decreased in the DENV-immune groups and modestly increased in the DENV-naïve group, resulting in a median proportion of cross-reactive MBCs of 26%, 36%, and 32.5% in the DENV-naïve/ZIKV, DENV-ZIKV, and +DENV-ZIKV groups, respectively (Fig. 3b).

When the frequency of the cross-reactive response was analyzed over total IgG-producing activated MBCs (ASC), we found a similar trend of significantly greater ZIKV and DENV cross-reactive responses at ~2 weeks postinfection in the DENV-ZIKV and +DENV-ZIKV groups (3.8% and 2.3%, respectively) compared to the DENV-naïve/ZIKV group (0.7%; *p* < 0.05, Kruskal–Wallis test and Dunn’s test) (Fig. 3c). The significantly higher magnitude of ZIKV and DENV cross-reactive responses was not sustained over time, and DENV-naïve, DENV-ZIKV and +DENV-ZIKV had a median proportion of 0.8%, 1%, and 1.2%, respectively, at ~8 months postinfection (Fig. 3d). The proportion of ZIKV and DENV cross-reactivity was significantly reduced over time in the DENV-ZIKV group (*p* < 0.05, Kruskal–Wallis test and Dunn’s test), while the reduction of cross-reactivity in the +DENV-ZIKV group was not significant (Supplementary Fig. 5).

### Previous DENV exposure number does not influence ZIKV MBCs

**Fig. 4:**
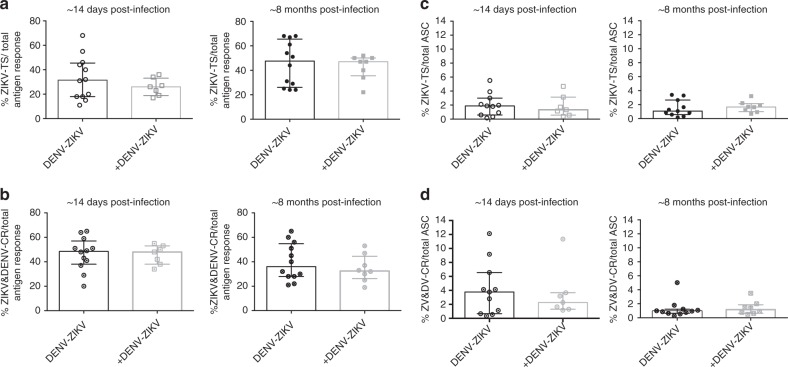
The number of previous DENV exposures does not affect type-specific and cross-reactive MBC responses. Comparison of ZIKV type-specific and ZIKV and DENV cross-reactive responses in ZIKV-infected patients with one (DENV-ZIKV) or more than one (+DENV-ZIKV) prior DENV exposure at ~14 days and ~8 months postinfection. **a** Proportion of ZIKV type-specificity (TS) of DENV-ZIKV and+DENV-ZIKV subjects over total antigen response. **b** Proportion of ZIKV & DENV cross-reactivity (CR) of DENV-ZIKV and +DENV-ZIKV subjects over total antigen response. **c** Frequency of type-specific responses of DENV-ZIKV and+ DENV-ZIKV subjects over total activated MBCs (ASC). **d** Frequency of cross-reactive responses of DENV-ZIKV and +DENV-ZIKV subjects over total ASC. The median and IQR are shown for all graphs. Statistical analysis was performed by Mann–Whitney test comparing DENV-ZIKV and +DENV-ZIKV at each time point, but no significant differences were found among different groups. DENV-naïve, *n* = 11; DENV-ZIKV, *n* = 12; +DENV-ZIKV, *n* = 7 at ~14 days postinfection (one PBMC sample was not viable), and *n* = 8 at ~8 months postinfection

In dengue-endemic areas, individuals can be exposed to more than one DENV infection, and neutralizing antibody responses become increasingly cross-reactive with iterative DENV infections^23^. Therefore, we investigated whether having one or more than one previous DENV infection would skew the MBC response following ZIKV infection to be more cross-reactive. Our data indicate that the average proportions of ZIKV type-specific and ZIKV-DENV cross-reactive responses at ~14 days and ~8 months postinfection are similar regardless of the number of prior DENV infections (Fig. 4a, b). The frequency of both type-specific and cross-reactive MBCs (among total ASC) was also similar between the two DENV-immune groups at both time-points assessed (Fig. 4c, d).

### Stronger ZIKV type-specific response in serum than in MBCs

**Fig. 5:**
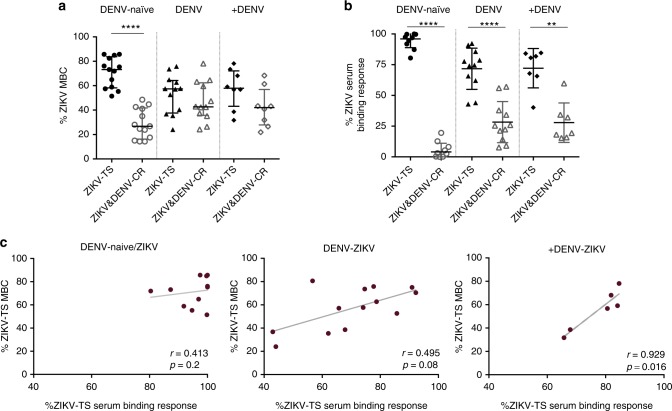
ZIKV infection elicits a strong type-specific binding response in serum antibodies. Analysis of the total ZIKV response in MBCs and serum from ZIKV-infected patients with no (DENV-naïve/ZIKV), one (DENV-ZIKV), or more than one (+DENV-ZIKV) prior DENV infection. **a** Proportion of ZIKV type-specific (TS) and cross-reactive (CR) responses over total ZIKV MBC response at ~8 months post-ZIKV infection. **b** Proportion of ZIKV type-specific (TS) and cross-reactive (CR) responses over total ZIKV serum antibody-binding response at ~8 months post-ZIKV infection. DENV-reactive antibodies were depleted from patient sera to obtain the proportion of the ZIKV response that is due to only ZIKV type-specific antibodies. **c** Correlation of the proportion of ZIKV TS responses in MBCs and serum. The median and IQR are shown for **a** and **b**. Significance was determined by Mann–Whitney test for **a** and **b**, and correlation analysis in **c** was performed using Spearman *r* test. *p < 0.05, **p < 0.01, ***p < 0.001. The sample size for each group is as follows: DENV-naïve/ZIKV MBC, *n* = 13 and serum *n* = 10; DENV-ZIKV, MBC *n* = 12 and serum *n* = 11; +DENV-ZIKV MBC *n* = 8 and serum *n* = 7

It remains unclear to what extent the specificities represented in the MBC pool parallel those of plasma cell-derived circulating antibodies following ZIKV infection and whether this varies based on prior DENV infection history. To directly compare the extent of cross-reactive binding in MBCs and serum antibodies, we studied both responses in the same individuals and measured their correlation ~8 months postinfection. For these experiments, only ZIKV-reactive MBCs were considered, and the proportion of ZIKV type-specific and cross-reactive MBCs within that population were determined (Fig. 5a). We found a strong ZIKV type-specific response in MBCs that remained dominant in the DENV-naïve/ZIKV group (median 73.2%, 95% CI: 63.6–78.4, *p* < 0.0001 compared to ZIKV and DENV cross-reactive responses, Mann–Whitney test) several months postinfection but that was compared to ZIKV and DENV cross-reactive responses in DENV-ZIKV (median 57.3%, 95% CI: 43–63.4) and +DENV-ZIKV (median 58%, 95% CI: 44.4–71.3) groups (Fig. 5a).

To examine serum antibody responses, DENV-reactive antibodies were depleted, and depletion was confirmed by elimination of DENV binding as measured by ELISA following depletion (Supplementary Fig. 6). The ZIKV type-specific binding antibody response was defined as the post-DENV depletion value divided by the value in control bovine serum albumin (BSA)-depleted serum. The difference between the ZIKV type-specific response and total response is designated the ZIKV and DENV cross-reactive antibody response (Fig. 5b). In serum, the anti-ZIKV binding antibody response was found to be highly type-specific regardless of DENV infection history: DENV-naïve/ZIKV (median 97.5%, 95% CI: 91.2–100.7, *p* < 0.0001, Mann–Whitney test), DENV-ZIKV (median 74.7%, 95% CI: 60.5–82.9, *p* < 0.0001, Mann–Whitney test) and +DENV-ZIKV (median 80.7%, 95% CI: 57.3–86.9, *p* < 0.001, Mann–Whitney test), where *p* values refer to percent of type-specific compared to percent of cross-reactive antibodies (Fig. 5b). Correlation analysis indicated no significant correlation between the proportion of ZIKV type-specific responses of MBCs with serum antibodies in the DENV-naïve/ZIKV group (*r* = 0.41; *p* = 0.2), a trending correlation in the DENV-ZIKV group (*r* = 0.5; *p* = 0.08), and a significant positive correlation for the +DENV-ZIKV group (*r* = 0.94; *p* < 0.05) (Fig. 5c).

### Type-specific antibodies dominate ZIKV neutralization

**Fig. 6:**
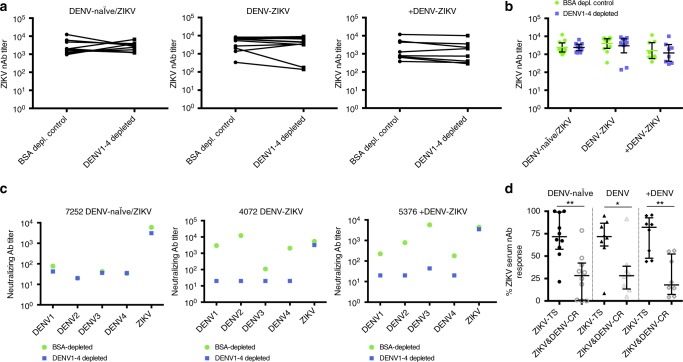
Depletion of DENV antibodies in serum does not have a significant effect on ZIKV neutralization. Neutralization analysis of DENV-depleted serum samples from ZIKV-infected patients with no (DENV-naïve/ZIKV), one (DENV-ZIKV), or more than one (+DENV-ZIKV) prior DENV infection. **a** ZIKV neutralizing antibody titer (FRNT_50_) in DENV-depleted versus control (BSA)-depleted samples for each group of ZIKV-infected patients at ~8 months postinfection. Lines connect results from each individual. **b** Statistical analysis of ZIKV neutralizing titers before and after depletion of DENV-reactive antibodies at ~8 months postinfection. Statistical analysis was performed by Wilcoxon test, but no significance differences were found among nondepleted and DENV-depleted ZIKV neutralizing antibody titers. **c** ZIKV neutralization antibody titers before and after depletion of DENV-reactive antibodies from three representative subjects analyzed in **a**. **d** Analysis of ZIKV type-specificity (TS) and ZIKV and DENV cross-reactivity (CR) in total ZIKV neutralizing response. Median and IQR is shown for **d**, and significance was determined by Mann–Whitney test. *p < 0.05, **p < 0.01, ***p < 0.001. Neutralizing antibody titers were log_10_ transformed for the analysis of **a**–**c**. DENV-naïve/ZIKV, *n* = 10; DENV-ZIKV, *n* = 10; +DENV-ZIKV, *n* = 8

To determine the contribution of type-specific versus cross-reactive antibodies to ZIKV neutralization capacity, serum samples collected ~8 months postinfection were analyzed before and after depletion of DENV-reactive antibodies to determine DENV and ZIKV neutralizing antibody titers (Supplementary Fig. 7). In the vast majority of cases, the neutralizing antibody titer against ZIKV was similar between DENV-depleted and control-depleted samples for each group (Fig. 6a–c), indicating that ZIKV type-specific antibodies account for the majority of neutralizing activity. We next analyzed the proportion of type-specific versus cross-reactive antibodies of the total ZIKV neutralizing antibody response. The ZIKV type-specific neutralizing antibody response was defined as the post-DENV depletion neutralization titer divided by the neutralization titer in control-depleted serum. The difference between the type-specific response and the total response is the ZIKV and DENV cross-reactive neutralizing antibody response (Fig. 6d). The type-specific fraction of ZIKV responses was significantly higher in the DENV-naïve/ZIKV (median 71%; *p* < 0.01, Mann–Whitney test), DENV-ZIKV (median 71%; p < 0.05, Mann–Whitney test) and +DENV-ZIKV groups (median 82%; *p* < 0.01, Mann–Whitney test) when compared to ZIKV and DENV cross-reactivity (Fig. 6d).

### ZIKV infection shows highest MBC cross-reactivity to DENV3

**Fig. 7:**
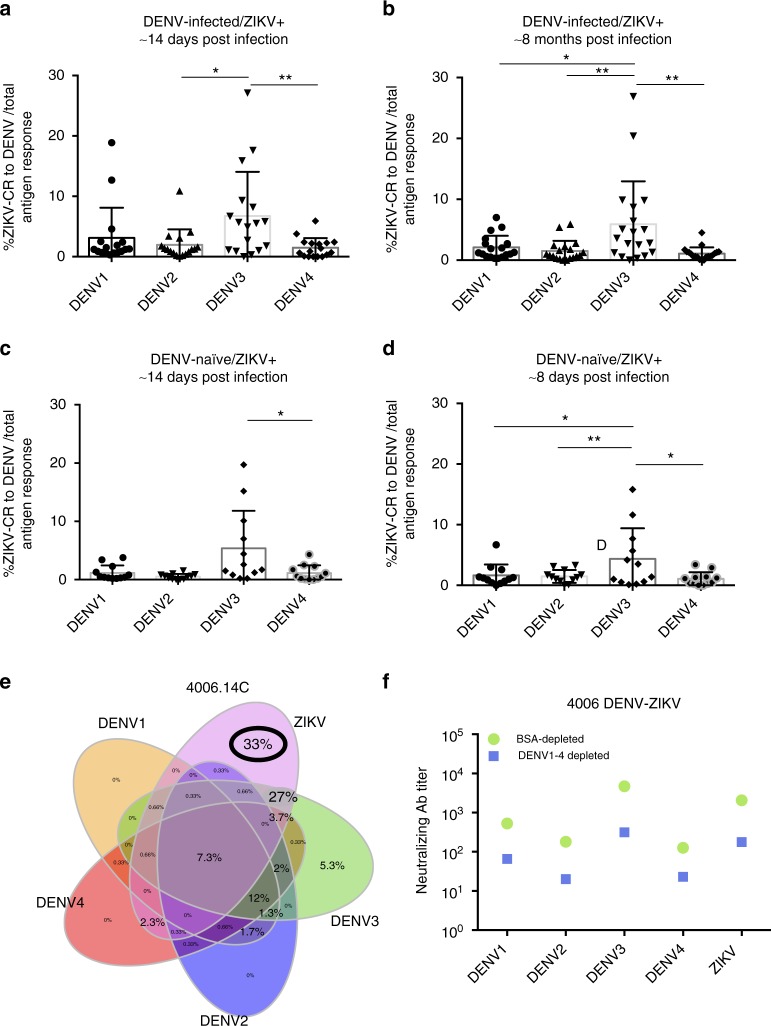
ZIKV infection displays highest cross-reactivity to DENV3. The MBC cross-reactivity between ZIKV and DENV was analyzed for each DENV serotype individually. The analysis was performed in the DENV-naïve/ZIKV+ and DENV-infected/ZIKV+groups. **a**, **b** Proportion of ZIKV cross-reactivity to DENV serotypes in DENV-infected/ZIKV + individuals at ~14 days (**a**) and ~8 months (**b**) post-ZIKV infection. **c**, **d** Proportion of ZIKV cross-reactivity to DENV serotypes in DENV-naïve/ZIKV + patients at ~14 days (**c**) and ~8 months (**d**) post-ZIKV infection. **e** Venn diagrams of type-specific and cross-reactive responses to ZIKV and DENV in MBCs of activated PBMCs from patient 4006 at ~8 months post-ZIKV infection. **f** DENV-depleted and nondepleted (BSA control) serum responses from patient 4006 at ~8 months post-ZIKV infection. The median and IQR are shown for **a**–**d**. Significance was determined by ordinary one-way Anova Multiple Comparison. *p < 0.05, **p < 0.01, ***p < 0.001. DENV-naïve/ZIKV+, *n* = 12; DENV-infected/ZIKV+, *n* = 17

ZIKV and DENV1–4 are phylogenetically related, but it is still unclear whether cross-reactivity with ZIKV may vary by DENV serotype. We tested for specific DENV serotype-ZIKV reactivity in ZIKV-infected patients who were DENV-naïve or had experienced prior DENV-infection(s). DENV-infected patients had been previously exposed to DENV1, DENV2, or DENV3 in similar numbers (*n* = 8, 9, 11, respectively) (Supplementary Fig. 8), and we analyzed them together as one group (DENV-infected). When comparing the median proportion of ZIKV cross-reactivity toward DENV1 (1.2%), DENV2 (1.2%), DENV3 (5.5%), and DENV4 (0.9%) at ~14 days postinfection in the DENV-immune/ZIKV group, we found that ZIKV and DENV3 cross-reactivity was significantly higher compared to ZIKV cross-reactivity with DENV2 and DENV4 (*p* < 0.05 and *p* < 0.01, respectively, one-way ANOVA test) (Fig. 7a). At ~8 months postinfection, the median proportion of ZIKV cross-reactivity toward DENV1 (1.3%), DENV2 (0.8%), DENV3 (3.6%), and DENV4 (0.8%) again indicated that ZIKV and DENV3 cross-reactivity was significantly higher compared to the ZIKV cross-reactivity toward the other three serotypes (*p* < 0.05, *p* < 0.01, *p* < 0.01 to DENV1, DENV2, DENV4, respectively, one-way ANOVA test) (Fig. 7b). Analysis of the DENV-naïve/ZIKV group at ~14 days postinfection showed a median proportion of ZIKV cross-reactivity with DENV1, DENV2, DENV3, and DENV4 of 0.5%, 0.6%, 2.2%, and 0.6%, respectively. ZIKV and DENV3 cross-reactivity was significantly higher compared to DENV4 cross-reactivity (*p* < 0.05, one-way ANOVA test) (Fig. 7c). After ~8 months postinfection, ZIKV cross-reactivity with DENV3 (2.5%) was again significantly higher when compared to DENV1 (1.1%), DENV2 (1.4%), and DENV4 (0.8%) cross-reactivity (*p* < 0.05, *p* < 0.01, *p* < 0.05, respectively, one-way ANOVA test) (Fig. 7d). Certain DENV-infected patients who presented strong cross-reactivity towards ZIKV and DENV3 (e.g., 27% in patient 4006) at the MBC level (Fig. 7e) also displayed a substantial decrease in ZIKV neutralizing titers when DENV-reactive antibodies were depleted, indicating a higher proportion of neutralizing activity attributable to cross-reactive antibodies (Fig. 7f).

## Discussion

To date, most ZIKV B cell studies have focused on panels of mAbs obtained at a single time-point after ZIKV infection from a small number of individuals without detailed knowledge of prior DENV infection history^25,32–35^. Recent advances have improved our understanding of the specificity and cross-reactivity of serum antibody responses to ZIKV and DENV^36,37^, but the relationship between serum antibodies and MBC specificity has not been studied, and a broader analysis of the polyclonal MBC response is lacking. Here, we performed a comprehensive assessment of MBC and serum polyclonal responses in 31 ZIKV+ pediatric patients with one or more well-characterized prior DENV infections as well as DENV-naïve patients from a cohort of children ongoing for 15 years. This allowed detailed analysis of the effect of prior DENV immunity on type-specific and cross-reactive MBC and serum antibody responses post-ZIKV infection.

The novel Multi-Color FluoroSpot assay allowed us to estimate the specificity of each MBC to DENV1-4 and ZIKV and the overall magnitude and proportion of MBCs that were reactive to DENV only, ZIKV only, or ZIKV and DENV. We found that ZIKV infection induced a strong type-specific MBC response in the presence or absence of prior DENV immunity that was maintained over time. Zika patients with one or more prior DENV infections exhibited a higher proportion of ZIKV/DENV cross-reactivity during early convalescence when compared to DENV-naïve patients, suggesting the activation of DENV immunological memory, consistent with previous publications^26,32,34^. However, over time, the magnitude and proportion of cross-reactive responses decreased in both DENV-ZIKV and +DENV-ZIKV groups and became similar to ZIKV type-specific responses. In contrast, the cross-reactivity of MBCs from DENV-naïve/ZIKV+ subjects increased slightly over time, similar to observations in primary DENV infections^39^, probably due to ongoing dynamics of selection, differentiation, and maintenance within the recently activated MBC population for months after infection. Our data suggest that ZIKV infection after prior DENV exposure engenders distinct MBC responses compared to a secondary DENV infection. Secondary ZIKV infection leads to a strong and enduring type-specific response, whereas in a secondary DENV infection, cross-reactive MBC responses developed during a primary DENV infection are expanded, maintained, and dominate over type-specific responses to the subsequently infecting DENV serotype, both at early and late convalescence^40–42^.

Our results suggest that MBC responses are more cross-reactive than serum antibodies. In both individuals who were DENV-naïve and DENV-immune, serum antibodies following ZIKV infection displayed a higher proportion of ZIKV type-specificity at the binding level than MBCs. Overall, serum antibody-binding responses to ZIKV were almost entirely driven by type-specific antibodies. In contrast, in MBC responses following ZIKV infection, DENV-naïve patients had a relatively greater ZIKV type-specific component, but still had a higher proportion of DENV-ZIKV cross-reactivity than was present in serum antibodies, while patients with prior DENV infection had similar proportions of type-specific and ZIKV/DENV cross-reactive MBC responses. These results suggest broader antigen specificity of ZIKV MBC responses in all patients as compared to serum antibodies, with greater cross-reactivity in the MBC pool in those with both DENV and ZIKV infection histories than those who had been infected with only ZIKV. Greater antigenic breadth in MBCs than LLPCs has been previously proposed in studies of West Nile virus infection in mice^15^^15^

Analysis of ZIKV type-specific responses in MBCs and serum overall showed no or a weak correlation in the DENV-naïve/ZIKV+ and DENV-ZIKV groups, respectively. A lack of correlation has also been observed between antibody levels in serum and peripheral MBCs in other viral infections and could be explained by the different roles proposed for MBCs and LLPCs^15,43^. A stronger correlation was observed for the +DENV-ZIKV patients, even though the sample size for this group was smaller, likely because serum antibodies were more cross-reactive after more than one DENV infection. The role of MBCs in protection or pathogenesis of secondary DENV infections is not yet fully understood. MBCs have been implicated as part of a strong plasmablast response correlated with severe DENV infection^44^, while a recent study showed down-regulation of plasmablast responses in subjects with asymptomatic DENV^45^. However, a diverse repertoire within the MBC pool could also be more likely to be rapidly activated during secondary infections with strains or serotypes that differ from the prior infecting pathogen and potentially play a protective role^15,46^. More studies simultaneously analyzing both LLPC- and MBC-derived antibodies are needed to clarify the contribution of MBCs to protection or pathogenesis of subsequent flaviviral infections.

Our specimens were collected from a long-term prospective cohort study in a dengue-endemic area where ZIKV was recently introduced. Before the Zika epidemic, no other flaviviruses were known to co-circulate with DENV in our study site. We had previously tested our cohort samples for West Nile Virus infection by neutralization assay, but did not find any evidence^47^. Yellow fever is not endemic in Nicaragua, and the vast majority of the population does not receive the yellow fever vaccine; no other flaviviruses have been reported in the country. Therefore, based on the documented DENV infection history of the children in the cohort study, we were able to analyze the impact of one or more prior DENV infections on cross-reactivity of the ZIKV/DENV MBC response. Interestingly, the number of prior DENV infections did not affect the magnitude or proportion of ZIKV type-specific or ZIKV/DENV cross-reactive MBC responses maintained into late convalescence. E protein structural features of the DENV serocomplex may play a role in restricting the breadth of antibody neutralization recognition to the DENV serocomplex. Other differences between the envelopes of dengue viruses and ZIKV, such as maturation state, flexibility and stability, could also play a role in exposure and immunogenicity of broadly conserved epitopes^48^.

In our study, analysis of ZIKV+ subjects from a dengue-endemic area who had been previously infected with DENV or who were DENV-naïve showed that ZIKV neutralization capacity was not substantially reduced after depletion of DENV-reactive antibodies, suggesting that type-specific antibodies are responsible for most of the neutralization of ZIKV in serum following natural infections. This is consistent with our preliminary observation that depleting DENV-reactive antibodies had no effect on ZIKV neutralizing activity in plasma of two DENV-immune returned travelers with ZIKV infection^36^. These findings are supported by previous studies of ZIKV/DENV cross-reactive mAbs showing poor cross-neutralization capacity against ZIKV and DENV^11,25,49^. In this regard, ZIKV may behave like other flaviviruses outside the DENV serocomplex, which are not effectively neutralized by antibodies induced in people exposed to one or more DENV infections. In humans, primary DENV infections stimulate high levels of type-specific neutralizing antibodies against the homologous serotype and lower levels of cross-reactive antibodies that do not contribute to type-specific neutralization^50^. Secondary DENV infections stimulate high levels of cross-reactive and cross-neutralizing antibodies against different serotypes with only small fractions of the overall response attributable to type-specific antibodies to the first and second infecting serotypes^23^. Altogether, our findings suggest that ZIKV as a secondary flavivirus infection after DENV infection behaves differently from a secondary DENV infection.

Similarly, antibodies from MBC-derived supernatants displayed high ZIKV binding titers and neutralizing titers, regardless of previous DENV immunity. These results reflect that, as found for serum antibodies, MBC-derived antibodies also have a strong neutralizing response against ZIKV. Even though MBC-derived supernatants contained high binding antibody titers against the different DENV serotypes, neutralizing antibodies to DENV were undetectable in most of the patients. This suggests that ZIKV infection generates MBC antibodies that at the functional level most strongly contribute to ZIKV neutralization. Overall, our results showed that MBCs, serum antibodies and MBC-derived antibodies developed post-ZIKV infection are dominated by a ZIKV-specific response.

Interestingly, a few of our patients did show a substantial reduction of ZIKV neutralization capacity after depletion of DENV-reactive antibodies. Specifically, we found higher cross-reactivity between ZIKV and DENV3, independent of previous DENV-immune status. Prior to the ZIKV outbreak in 2016 in the Nicaraguan PDCS, DENV2 was the dominant circulating serotype from 2014 to 2016, preceded by DENV1 from 2012 to 2013, and DENV3 from 2009 to 2011^51^. It could be that one or a few highly immunogenic epitopes are conserved between DENV3 and ZIKV. Our patients represented a spectrum of DENV1, DENV2, and DENV3 infections prior to ZIKV in roughly equal numbers, yet DENV3/ZIKV cross-reactivity was the highest observed, including in DENV-naïve Zika cases. This argues that this cross-reactivity is not simply being driven by a predisposition to cross-react with the most recent infecting DENV serotype but rather perhaps by a conserved immunogenic epitope; however, this needs to be further investigated. Similarly, another study showed cross-reactivity between ZIKV and DENV1 from expanded MBC clones, targeting the EDIII lateral ridge^32^. This cross-reactivity was associated with high neutralizing responses to ZIKV^32^. Thus, prior DENV immunity in humans may have the potential to boost ZIKV responses to conserved epitopes, and ZIKV infection alone may have specific cross-reactive epitopes with some, but not all, DENV serotypes. Further studies are needed to elucidate the identity of cross-reactive epitopes shared by ZIKV and DENV3 or other DENV serotypes.

In addition to the crucial role in durable immunity to ZIKV played by antibody and MBC responses, several mouse studies have shown a protective role for T cell responses against ZIKV infection. CD4^+^ and CD8^+^ T cells were found to reduce viral load and severe disease outcomes during ZIKV infection^52–54^. CD4^+^ T cells were shown to help induce an effective neutralizing antibody response to control ZIKV replication in interferon receptor-deficient mice^53^. Moreover, cross-reactive CD8^+^ T cells from prior DENV infection can protect against ZIKV infection during pregnancy in mice^55^. Human studies have shown that prior DENV exposure influences the timing, magnitude, and quality of the T-cell response during ZIKV infection^56^; nevertheless, it is still unknown whether cross-reactive T cells have a protective role during ZIKV infection.

This research has several strengths. We have analyzed B cell responses to ZIKV in a larger number of individuals than previously reported, who derive from a long-term pediatric dengue and Zika cohort in an arbovirus-endemic setting. The cohort study design allows for the documentation of symptomatic and inapparent DENV infection history of the participants over many years. This allowed us to use a set of well-characterized samples with different prior DENV infection history before the ZIKV epidemic. Serum and MBC analyses from all the patients were analyzed in parallel at a polyclonal level, differing from previous studies that have mainly focused on analysis of mAbs. Furthermore, the neutralization and binding capacity of antibodies deriving from different subsets of B cells (e.g., MBCs and plasma cells) were studied for their functional and binding capacities. Finally, we use a novel Multi-Color FluoroSpot assay to examine the type-specificity and cross-reactivity of ZIKV infection to the different DENV serotypes.

Our study has some limitations. Because the cell number was limited in samples from our pediatric study population, we were only able to perform limited functional analyses to measure neutralization of MBC-secreted antibodies. However, we did perform antibody depletions and neutralization assays of serum antibodies from the same subjects. Additionally, our study focused on the investigation of B cell responses following ZIKV infection in DENV-naïve or DENV-immune individuals. We were not able to study DENV infections in ZIKV-immune subjects, because no DENV infections following ZIKV infection have yet been observed in the cohort. Such studies are still needed to address ZIKV and DENV cross-reactivity in DENV-infected subjects with prior ZIKV infection.

The role of B cells during ZIKV infection is still not completely understood, and the circulation of ZIKV in dengue-endemic areas has elicited interest and concern regarding the effect of cross-reactive responses on protection or exacerbation of disease in both ZIKV and DENV infections. Although ZIKV is phylogenetically closely related to DENV, our results show that ZIKV generates a B cell and antibody response that has a strongly type-specific component even in individuals with prior DENV immunity. These findings reveal that pre-existing immunity in subjects who had experienced 1 or >1 prior DENV infections only modestly shapes the breadth and magnitude of enduring ZIKV-reactive MBC responses to ZIKV and does not preclude de novo development of ZIKV-specific antibody responses from naïve B cells. This suggests that a ZIKV vaccine in DENV-immune individuals would still engender type-specific neutralizing antibodies to ZIKV. However, after ZIKV infection, both those with and without prior DENV infections maintain a substantial subset of MBCs and antibodies that are DENV-ZIKV cross-reactive, suggesting possible modulation of the MBC and serum antibody response to DENV infection. This observation has important implications for protection against or enhancement of subsequent dengue disease. Overall, our study provides critical insights into cross-reactive and type-specific MBC and serum antibody responses to DENV and ZIKV in patients with different DENV infection histories, with implications for natural infections and vaccine development in areas where ZIKV and DENV co-circulate.

## Methods

### Ethics statement

The Pediatric Dengue Cohort Study (PDCS) was reviewed and approved by the Institutional Review Boards (IRBs) of the University of California, Berkeley, and the Nicaraguan Ministry of Health. Parents or legal guardians of all subjects provided written informed consent, and subjects 6 years of age and older provided oral assent. The protocol was amended in July 2015 to include screening for ZIKV infection in participants meeting the study case definition and again in February 2016 to expand the case definition (see below). These amendments were approved by the IRBs reviewing the study.

### Study design and population

PBMCs were obtained between July 2016 and March 2017 from ZIKV-infected patients who were DENV-naïve (*n* = 11) or who had previously been exposed to DENV once (*n* = 12) or more than once (*n* = 8) (Table 1) in the community-based prospective Pediatric Dengue Cohort Study (PDCS) of ~3700 children that has been ongoing since 2004 in Managua, Nicaragua^57^. Blood samples were collected from suspected Zika cases who presented with rash with one or more of the following: conjunctivitis, arthralgia, myalgia, and/or peri-articular edema, regardless of fever^58^. ZIKV infection was confirmed by real-time RT-PCR performed at the National Virology Laboratory of the Ministry of Health in Managua using either of two triplex assays that simultaneously detect ZIKV, CHIKV, and DENV infections: the ZCD assay^5,59^ or the CDC Trioplex assay^60^. Samples from ZIKV-positive patients were collected at early (~14 days postinfection) and late (~8 months postinfection) convalescence (Table 1). The definition of convalescence follows the norm for the 15 years of our cohort study and the convention in the dengue field; namely, early convalescent sample collected 14–21 days postonset of illness^61–64^ and late convalescent >6 months postillness^57^^57^

Confirmed ZIKV-positive cases were classified as DENV-naïve if they entered the cohort study with no detectable anti-DENV antibodies, as measured by the DENV Inhibition ELISA (IE) assay, which has a sensitivity of 98.9% compared to the Hemagglutination Inhibition assay^21,47^, and had no documented DENV infections (symptomatic or inapparent) during their time in the cohort. Confirmed ZIKV-positive cases were classified as DENV-immune if they entered the cohort study with no detectable anti-DENV antibodies and had one (DENV-ZIKV) or more than one (+DENV-ZIKV) documented DENV infections during their time in the cohort. The previous DENV infection history of patients who tested positive by ZIKV RT-PCR was initially determined by testing annual longitudinal samples collected before the introduction of ZIKV to Nicaragua. Subjects who seroconverted or displayed a ≥fourfold increase in antibody titer when pre-ZIKV annual samples were tested side-by-side using the DENV Inhibition ELISA assay^21,47^ were determined to have had at least one previous DENV infection. Subsequently, longitudinal samples collected annually from before the first DENV infection through two years after the last DENV infection before the ZIKV infection for each individual were analyzed by a flow-based neutralization assay to confirm the number of DENV infections experienced before the ZIKV infection^65,66^.

### PBMC isolation

For PBMC preparation^41,67^, blood samples were collected in Vacutainer tubes (Becton–Dickenson) with 5 mM EDTA as anticoagulant. Upon receipt at the Nicaraguan National Virology Laboratory, ~5 ml of blood was transferred into a Leucosep tube (Greiner Bio-One) containing 3 ml of Ficoll Histopaque (Sigma) and centrifuged at 500*g* for 20 min (min) at room temperature. The PBMC fraction was collected and transferred to a tube containing 9 ml of PBS with 2% fetal bovine serum (FBS; Denville Scientific) and 1% penicillin/streptomycin (Sigma). Cells were washed and pelleted three times by centrifugation at 500*g* for 10 min and resuspended in RPMI 1640 complete medium (RPMI 1640, 10% FBS, 1% GlutaMAX^™^, 1% HEPES and 1% penicillin/streptomycin). Before the third wash, cells were counted using a hemocytometer (Sismex XS-1000i). After the third wash, cells were resuspended in cryovials at a concentration of 3 × 10^6^ cells/ml in freezing medium (90% FBS, 10% dimethyl sulfoxide) and were placed in isopropanol containers (Mr. Frosty, Nalgene) at −80 °C overnight and transferred to liquid nitrogen for storage^41,67^.

### Virus propagation and purification

*Aedes albopictus* C6/36 cells, kindly donated by R. Baric (University of North Carolina at Chapel Hill), were grown to 70% confluence in a 175 cm^2^ vented tissue culture flask in MEM medium (Life Technologies) supplemented with 10% FBS (Corning), 1% GlutaMAX^™^ (Life Technologies), 1% HEPES (Life Technologies), and 1% penicillin/streptomycin (Sciencell). The cells were then infected with unconcentrated ZIKV or DENV1–4 for 5 days. The infectious supernatant was concentrated using a 100-kDa Amicon filter tube (Millipore) and then layered on a discontinuous 20/55% OptiPrep^®^ (Sigma-Aldrich) density gradient and ultracentrifuged at 40,000 RPM at 4 °C for 2 h using a SW41Ti rotor, without brakes. Finally, the purified virus was collected from the interface between 20 and 55% density and was stored at −80 °C.

### Virus quality control

Viral RNA was extracted from unconcentrated virus using a QIAmp^®^ Viral Mini Kit (Qiagen), and the concentration was determined using a NanoDrop^®^ Spectrophotometer. To determine the serotype of the virus, RNA was used in a SuperScript^®^ One-Step RT-PCR with Platinum^®^ *Taq* (Invitrogen) assay with the appropriate primers to amplify ZIKV and the four DENV serotypes. Protein concentration of purified DENV and ZIKV was determined using a Pierce^TM^ BCA Protein Assay Kit (Thermo Scientific). Purified virus was tested for serotype-specificity by indirect ELISA.

### ELISA

Prior to Qdot conjugation, serotype specificity of the detection mAbs (anti-DENV1 E95, anti-DENV2 E96, anti-DENV3 5J7, anti-DENV4 E88, and anti-ZIKV ZKA64) was assessed by indirect ELISA^38^. Briefly, plates were coated with a dilution of OptiPrep-purified DENV and ZIKV (10 μg/ml) in coating buffer at 37 °C for 2 h, followed by 5 washes with PBS 0.05% Tween (PBS-T), then plates were blocked with 5% milk in PBS-T for 2 h at 37 °C. Detection mAbs were incubated at a 1:100 dilution in blocking buffer at 37 °C for 2 h. After five washes with PBS-T, the plates were incubated with either anti-human horseradish peroxidase (HRP) (Jackson ImmunoResearch) (1:1000) or anti-mouse-HRP (Jackson ImmunoResearch) (1:500) mAbs at 37 °C for 2 h. Plates were then washed five times with PBS-T and visualized with 3,3′,5,5′-tetramethylbenzidine; the reaction was stopped with sulfuric acid after 10 min. To ensure serotype specificity, each mAb was tested against all the different viruses.

Confirmation of DENV antigen depletion was performed by ELISA by directly coating ELISA plates with purified DENV antigen (100 ng/well), followed by blocking with 3% nonfat dairy milk (LabScientific, Inc.) and incubating with sera for 1 h at 37 °C. IgG-binding antibodies were detected with an alkaline phosphatase-conjugated goat anti-human secondary antibody (Sigma) and ρ-nitrophenyl phosphate substrate (Sigma). Absorbance was measured at 405 nm using an Epoch plate reader (BioTek). For the ZIKV-IgG binding ELISA, plates were coated with a cross-reactive DENV/ZIKV binding antibody (4G2, 100 ng/well) and blocked with 3% nonfat milk. The plates were then incubated with ZIKV antigen (stock grown in C6/36 cells and diluted 1:1 in blocking buffet) for 1 h at 37 °C. IgG-binding antibodies were detected as described above.

### Conjugation of mAbs to Qdots

After confirmation of mAb quality and specificity, the different mAbs were conjugated to dibenzocyclooctynol (DIBO)-modified Qdot^®^ nanocrystals. SiteClick^TM^ technology was used to conjugate Qdots (Life Technologies) directly to the Fc region of purified mAbs (100–125 µg), according to the manufacturer’s instructions. We used Qdot^®^525, Qdot^®^625, Qdot^®^700, and Qdot^®^800 to conjugate four of the five mAbs with this protocol. To label the fifth mAb, we used the EZ-Link sulfo NHS LC Biotinylation kit (ThermoFisher Scientific) to conjugate several biotin molecules to amino groups on the mAbs. The biotinylated mAbs were then detected with Qdot 565-labeled Streptavidin.

### Multi-Color FluoroSpot

The Multi-Color FluoroSpot^38,68^ was performed by activating 96-well IPFL FluoroSpot plates (Cellular Technology Limited) with 70% EtOH, washing three times, and coating with 2 μg/well of Fcγ fragment-specific anti-human IgG (Jackson ImmunoResearch). Following overnight incubation at 4 °C, wells were blocked with RPMI 1640 complete culture medium at 37 °C for 2 h. Previously, PBMCs from ZIKV-infected subjects were thawed in RPMI 1640 complete culture medium with 1 μl/mL DNaseI and activated with IL-2 (1000 U/mL) and R848 (2.5 μg/μl) for 5 days. Activated PBMCs were counted and plated on the coated wells in eight 2-fold serial dilutions, following the blocking step. Due to the potentially low number of antigen-specific cells expected, activated PBMCs were plated starting with 2.5 × 10^5^–1 × 10^6^ PBMCs in the first well. After 48 h of incubation at 37 °C, PBMCs were removed via 3 washes with PBS-T, and the captured Abs were incubated with all five Optiprep^®^-purified antigens (20 μg/ml in 1× PBS) for 1 h at 37 °C. After three washes with PBS-T, wells were blocked with human gamma globulin (Jackson ImmunoResearch) diluted 1:500 in 1× DPBS, followed by incubation with Qdot-labeled mAbs diluted 1:1000 in DPBS 1× for 1 h at 37 °C. Finally, plates were washed 3 times with PBS-T and once with ddH_2_O and dried using a 96-well MultiScreen Vacuum Manifold (Millipore). The MBCs that reacted with the antigen(s) were visualized as spots representing single cells using a CTL ImmunoSpot S6 Micro-Analyzer (Cellular Technology Limited). Cross-reactive pan-DENV and ZIKV antibody EBV-transformed B cells were used as positive controls. The software was configured by Cellular Technology Limited (CTL) to count high numbers of spots in one well. The dilution at which ZIKV and DENV antigen-specific spots were clearly countable was used. For DENV-naïve ZIKV+  patients, it was necessary to count at a lower dilution of cells in order to detect sufficient numbers of DENV cross-reactive spots. At lower dilutions, ZIKV spots were more abundant, but as discussed above, the software was able to count high numbers of spots accurately.

To determine total IgG-secreting cells, PBMCs were plated in 1:2 serial dilutions of the stimulated cells starting from 5 × 10^5^ PBMCs/well. Wells were coated with Fcγ fragment-specific anti-human IgG. Following cell incubation and Ab deposition, IgG-positive spots were visualized using a polyclonal PE-labeled anti-human IgG detection mAb (H + L). Spots were then counted using the CTL reader.

### Flow cytometry-based neutralization assay

A flow cytometry-based neutralization assay using reporter virus particles (RVPs) was performed^65,66^. Briefly, in a 96-well round-bottom plate (Corning), a 1:5 dilution of sera and RPMI 1640 complete medium (pH 8.0) was added to all wells in the first row (A). Subsequently, a threefold serial dilution was performed, from row A down the plate. The RVP master mix was diluted to generate 7–20% infection and added in a 1:1 volume to all the wells, except the negative control, resulting in a 1:10 dilution in Row A. After the incubation period, 40,000 of Raji-DC-SIGN cells in RPMI 1640 complete culture medium were added to each well. After a 48-h incubation at 37 °C in 5% CO_2_, the cells were fixed with 2% formaldehyde (16% formaldehyde, Thermo Scientific) diluted in fluorescence-activated cell sorting (FACS) Buffer (1× DPBS supplemented with 0.5% BSA [RMBIO^®^] and 0.02% sodium azide [Sigma Aldrich]). Following fixation for 30 min in the dark at room temperature, the fixing solution was decanted and FACS buffer was added to each well for analysis by flow cytometry.

### Depletion of DENV-binding antibodies

A cross-reactive DENV-binding antibody (1M7) was conjugated to M280 Tosylactivated Dynabeads (Life Technologies) at a ratio of 100 μg 1M7 to 5 mg Dynabeads as per the manufacturer’s instructions. Beads were blocked with 1% BSA/PBS for 1 h at 37 °C. Purified DENV antigen was harvested from infected Vero cell supernatants then concentrated by tangential flow ultracentrifugation using the Pellicon mini system with a 100 kD cutoff membrane (Millipore), flow rate of 400 mL/min, filtration rate of 100 min/mL, and pressure of 20–30 psi. Using a 15–65% sucrose gradient, the concentrated virus was purified by ultracentrifugation (SW 40 Ti, Beckman Coulter) at 21,538 rcf for 18 h at 4 °C. The fractions were run on sodium dodecyl sulfate polyacrylamide gel electrophoresis (SDS-PAGE), and concentrations were then measured by Micro BCA Protein Assay Kit (Thermo Fisher Scientific). Purified DENV was bound to the 1M7-Dynabead mixture at a ratio of 100 μg antigen to 5 mg DENV-bound Dynabeads for 1 h at 37 °C and fixed using 2% paraformaldehyde. Control beads were incubated with an equal amount of BSA. For depletion, sera were diluted 1:10 and incubated with 5 μg of DENV-bound Dynabeads in 3 successive rounds at 37 °C for 1 h each. Following incubation, beads were removed from the serum using a magnet. Confirmation of DENV antigen depletion was performed by ELISA (see above).

### Focus-reduction neutralization test

Post-depletion neutralization titers were determined using a focus-reduction neutralization test (FRNT) in which serially diluted sera were incubated with 50–100 focus forming units of DENV1 (West-Pac ’74), DENV2 (S-16803), DENV3 (CH53489), DENV4 (TVP-376), and ZIKV (H/PF/2013) in Dulbecco modified Eagle medium with 2% FBS for 1 h at 37 °C. The serum-virus mixtures were then added to a monolayer of Vero cells in a 96-well plate and incubated for an additional 1 h at 37 °C. After infection, the cells were washed with 1× PBS and overlaid with Opti-MEM (Gibco) with 2% FBS and 1% (wt/vol) carboxymethyl cellulose (Sigma). Infected cells were incubated for 2 days at 37 °C and 5% CO_2_, fixed with paraformaldehyde, permeabilized, blocked with 3% nonfat dairy milk (LabScientific, Inc.), and stained with cross-reactive DENV antibodies (4G2 and 2H2). Wells were then incubated with a HRP-conjugated goat anti-mouse secondary antibody (KPL) and developed with TrueBlue peroxidase substrate (KPL)

### Statistical analysis

FluoroSpot results were analyzed with a Kruskal–Wallis test and Dunn’s test corrected for multiple comparisons of non-matched and nonparametric data using Prism 6 software (GraphPad Software). FluoroSpot results were also analyzed using a two-tailed Wilcoxon test and Mann–Whitney test for paired comparisons of nonparametric data using Prism 6 software. For depletion analysis, ELISA binding results were analyzed using a two-way ANOVA test with Sidak’s multiple comparison test. For neutralization assays, serum concentrations were log_10_ transformed and plotted using the sigmoidal dose response (variable slope) equation, and FRNT_50_ values were calculated. FRNT_50_ values from each sample were compared and analyzed using a two-way ANOVA with Sidak’s multiple comparison test. Correlation analysis of MBCs and serum reactivity was performed using a Spearman *r* test and a nonlinear fit regression analysis with Prism 6 software.

### Reporting summary

Further information on experimental design is available in the Nature Research Reporting Summary linked to this article.

## Supplementary information

Supplementary Information

Reporting Summary

## Data Availability

All main data supporting the findings are available within the article or the Supplementary Information. Other data are available from the authors upon request.
