# Supplementary material for: Impact of pre-existing dengue immunity on human antibody and memory B cell responses to Zika
Source: Nat Commun. 2019 Feb 26;10:938. doi: 10.1038/s41467-019-08845-3 (PMC6391383; doi:10.1038/s41467-019-08845-3)
Supplement: Supplementary file 1 — Supplementary Information [file 41467_2019_8845_MOESM1_ESM.pdf]

Impact of pre-existing dengue immunity on human antibody and memory B cell responses to Zika

Andrade et al.

Supplementary Materials

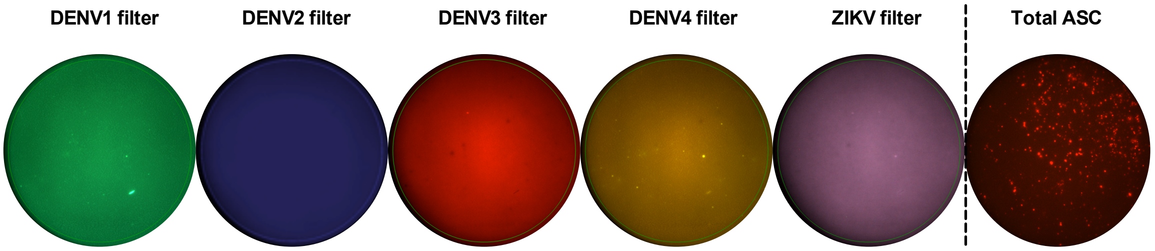

Supplementary Figure 1. Representative images of activated MBCs from patient 5072 (DENV-naïve and ZIKV-naïve). The antigen specificity of MBC responses to ZIKV and the four DENV serotypes is shown as spots represented in each filter. The total number of activated MBCs was analyzed in parallel and defined as antibody secreting cells (Total ASC). The different DENV and ZIKV filters do not show the presence of positive spots for any of the antigens, whereas the Total ASC well shows the expected activation of MBCs from patient 5072.

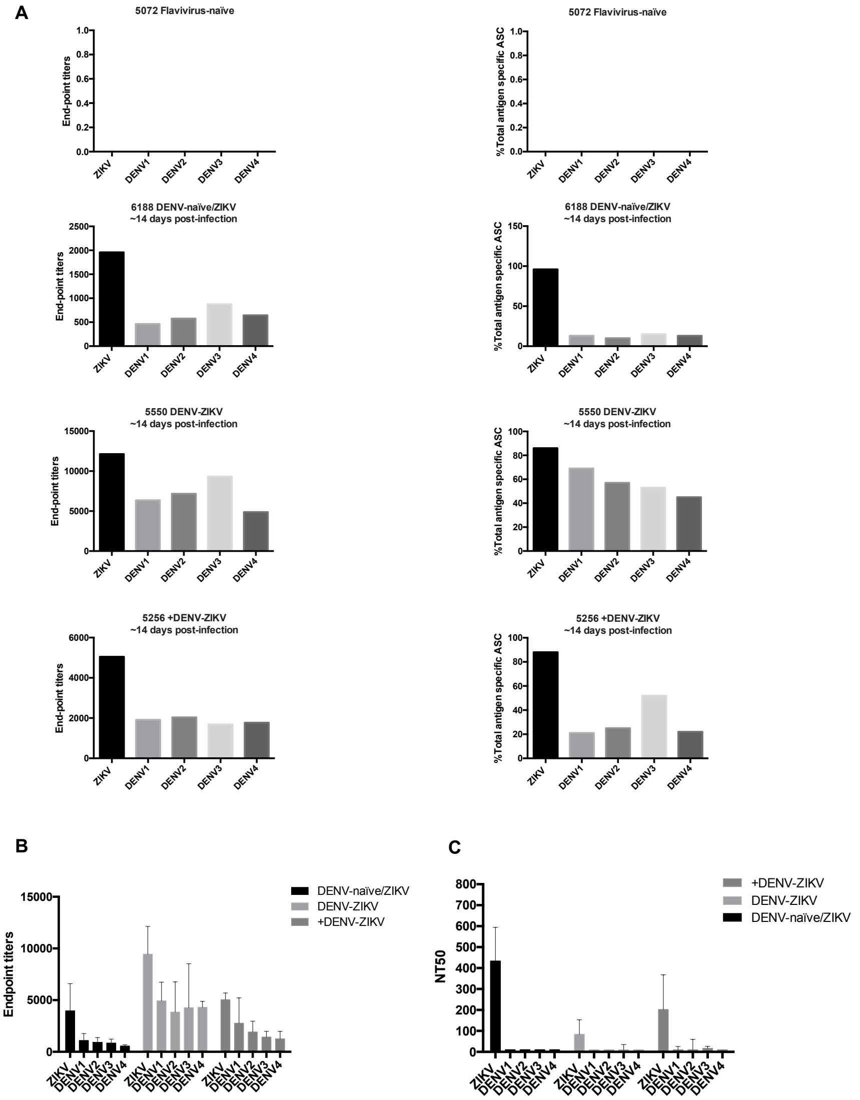

Supplementary Figure 2. Analysis of MBC-derived antibodies in ZIKV-infected+ patients at ~14 days post-infection. A. Antibodies from antigen-reactive MBCs to ZIKV, DENV1, DENV2, DENV3 and DENV4 in representative patients from the DENV-naïve/ZIKV, DENV-ZIKV, and +DENV-ZIKV groups at ~14 days post-infection, as well as flavivirus-naïve subjects, were measured in the supernatant of activated MBCs by antibody titration by ELISA (left panel) and compared to the reactivity of the MBCs from which the antibodies were derived using the Multi-color FluoroSpot (right panel). **B.** Antibodies from antigen-reactive MBCs to ZIKV, DENV1, DENV2, DENV3 and DENV4 in 12 patients from the DENV-naïve/ZIKV, DENV-ZIKV, and +DENV-ZIKV groups were measured in the supernatant of activated MBCs by antibody titration by ELISA. **C.** Neutralizing antibody titers (NT_{50}) to ZIKV and DENV1-4 in patients from the DENV-naïve/ZIKV, DENV-ZIKV, and +DENV-ZIKV groups measured in the supernatant of activated MBCs. Error bars indicate the SEM.

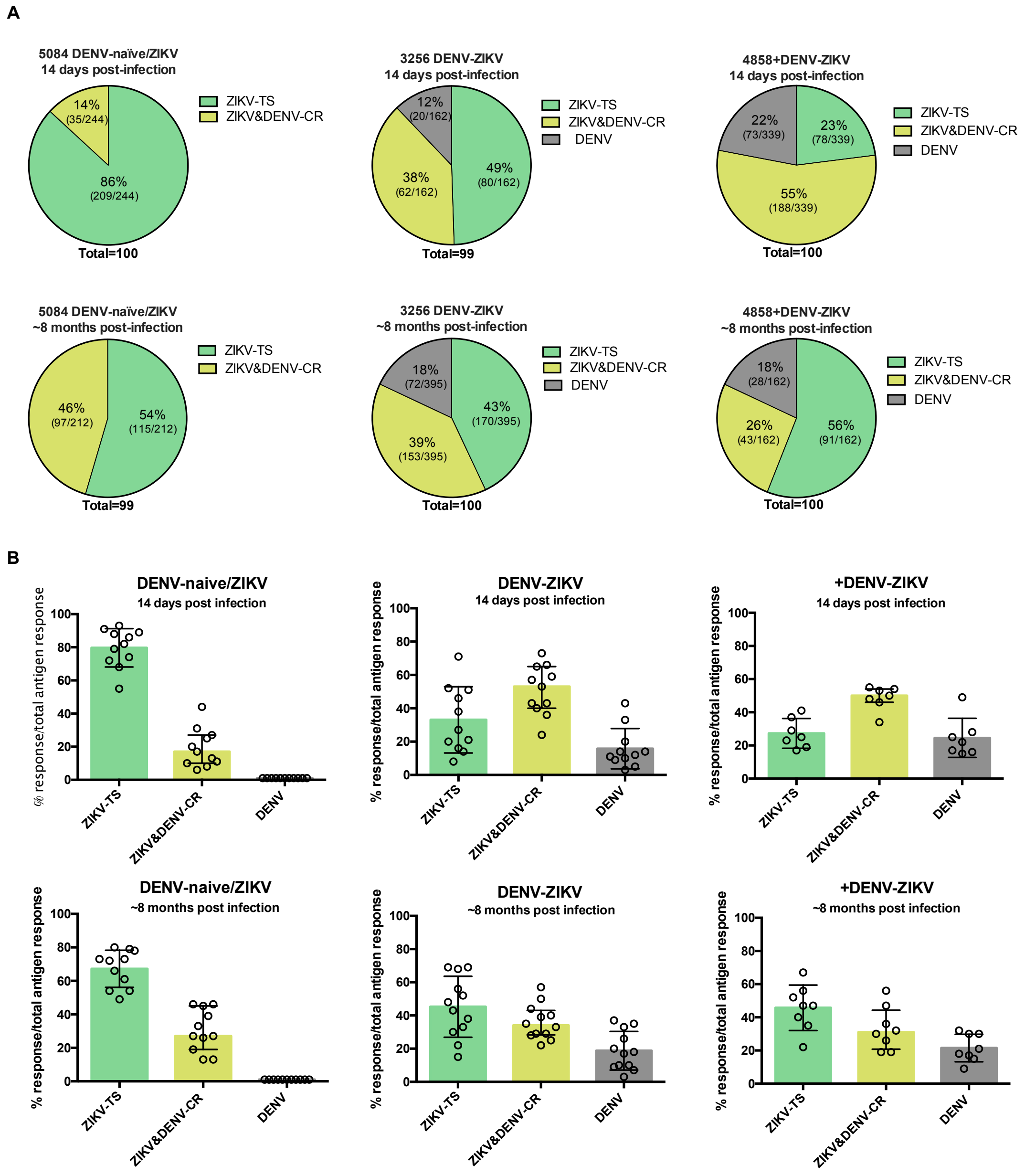

Supplementary Figure 3. MBC responses in ZIKV-infected+ patients at ~14 days and ~8 months post-infection. MBC responses in patients with RT-PCR-confirmed ZIKV infection and no (DENV-naïve/ZIKV), one (DENV-ZIKV), or more than one (+DENV-ZIKV) prior DENV infection were analyzed by the Multi-color Fluorospot assay. MBC responses recognizing only ZIKV were designated type-specific and labeled as ZIKV-TS. MBCs that recognized ZIKV and at least one DENV were considered cross-reactive and labeled as ZIKV&DENV-CR. MBC that recognized DENV but not ZIKV were considered DENV-specific and labeled as DENV. **A.** Proportion of antigen-reactive MBCs to ZIKV, ZIKV and DENV, and DENV in representative patients from the DENV-naïve/ZIKV, DENV-ZIKV, and +DENV-ZIKV groups at ~14 days and ~8 months post-infection. **B.** Proportion of antigen-reactive MBCs to ZIKV, ZIKV and DENV,

and DENV in patients from the DENV-naïve/ZIKV, DENV-ZIKV, and +DENV-ZIKV groups at ~14 days and ~8 months post-infection. The median and IQR are shown for all graphs. The sample size of each the group is as follows: DENV-naïve/ZIKV, n=11; DENV-ZIKV, n=12; +DENV-ZIKV, n=7 at ~14 days post-infection (one PBMC sample was not viable, n=7) and n=8 at ~8 months post-infection.

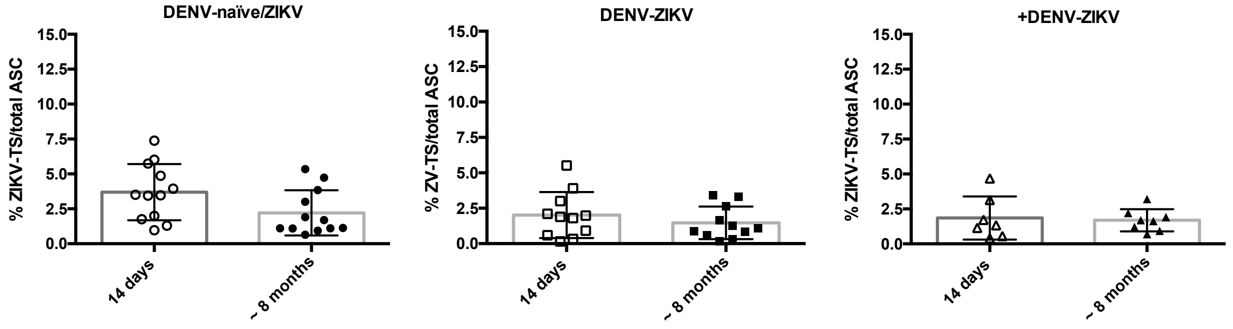

Supplementary Figure 4. **Comparison of the magnitude of ZIKV type-specific B cell responses in ZIKV-infected patients at ~14 days and ~8 months post-infection**. The magnitude of ZIKV type-specific B cell responses was measured out of total IgG-producing activated MBCs. The median and IQR are shown for all graphs. Statistical analysis was performed by Wilcoxon test, but no significance differences were found. DENV-naïve/ZIKV, n=11; DENV-ZIKV, n=12; +DENV-ZIKV, n=7 at ~14 days post-infection (one PBMC sample was not viable, n=7) and n=8 at ~8 months post-infection.

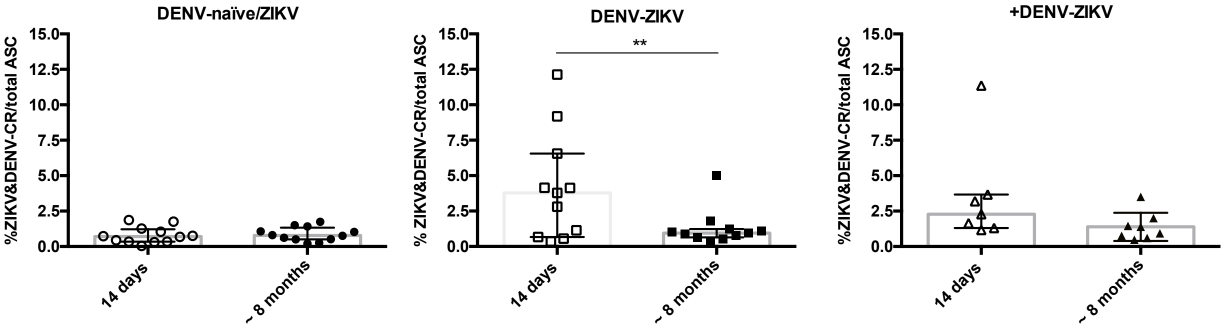

Supplementary Figure 5. Comparison of the magnitude of ZIKV and DENV cross-reactive B cell responses in ZIKV-infected patients at ~14 days and ~8 months post-infection. The magnitude of ZIKV type-specific B cell responses was measured out of total IgG-producing activated MBCs. The median and IQR are shown for all graphs. Significance was determined by Wilcoxon test *p<0.05, **p<0.01, ***p<0.001. DENV-naïve/ZIKV, n=11; DENV-ZIKV, n=12; +DENV-ZIKV, n=7 at ~14 days post-infection (one PBMC sample was not viable, n=7) and n=8 at ~8 months post-infection.

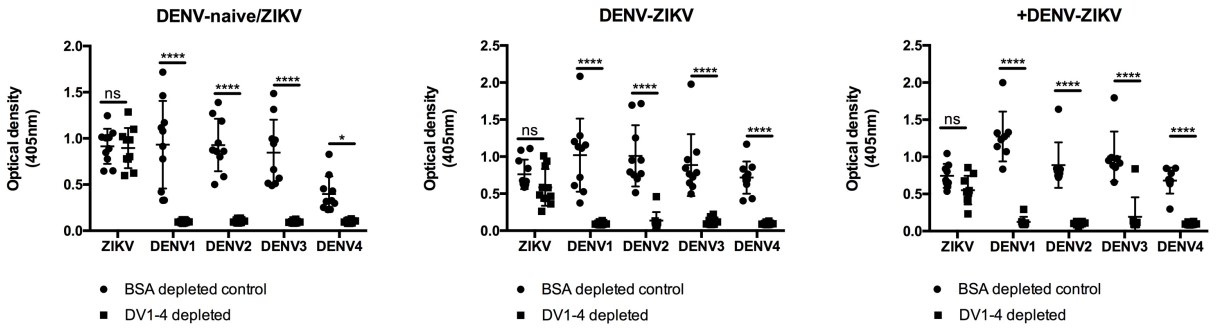

Supplementary Figure 6. Depleting with DENV antigen greatly reduces DENV- but not ZIKV-binding IgG antibodies in serum. Antigen-capture ELISA was performed to detect IgG binding to the viral antigen (indicated on the x-axis) in serum depleted of DENV-reactive antibody vs. control depletion for sera from DENV-naïve/ZIKV, DENV-ZIKV and +DENV-ZIKV patients. Optical density at 405nm is shown on the y-axis. The median and IQR are shown for all graphs. Significance was determined by a 2-way ANOVA with Sidak's multiple comparisons test. *$p<0.05$, **$p<0.01$, ***$p<0.001$, ****$p<0.0001$. DENV-naïve/ZIKV, n=10; DENV-ZIKV, n=9; +DENV-ZIKV, n=8.

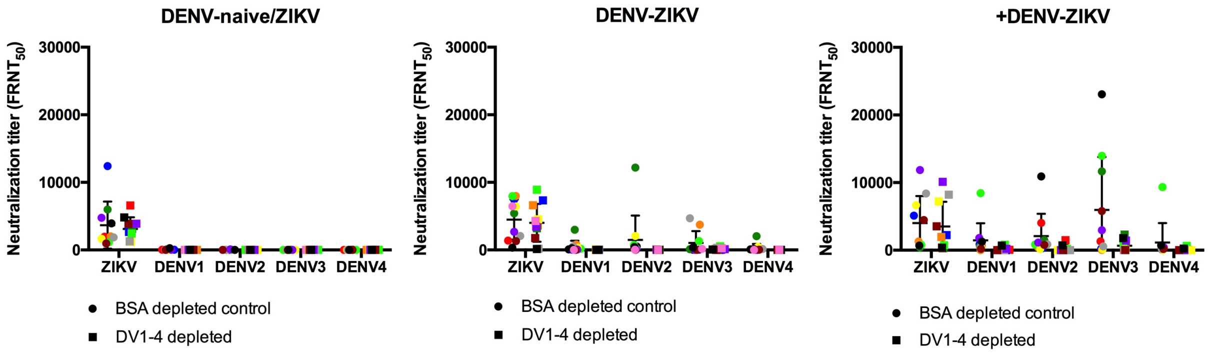

Supplementary Figure 7. **Analysis of neutralizing antibodies in serum samples depleted of DENV-reactive antibodies in ZIKV+ patients at ~8 months post-ZIKV infection.** Serum samples were depleted with BSA (control) or DENV-1-4 and then tested for neutralizing activity ($FRNT_{50}$) against ZIKV and the four DENV serotypes by FRNT assay. The median and IQR are shown for all graphs. DENV-naïve/ZIKV, n=10; DENV-ZIKV, n=9; +DENV-infected, n=8.

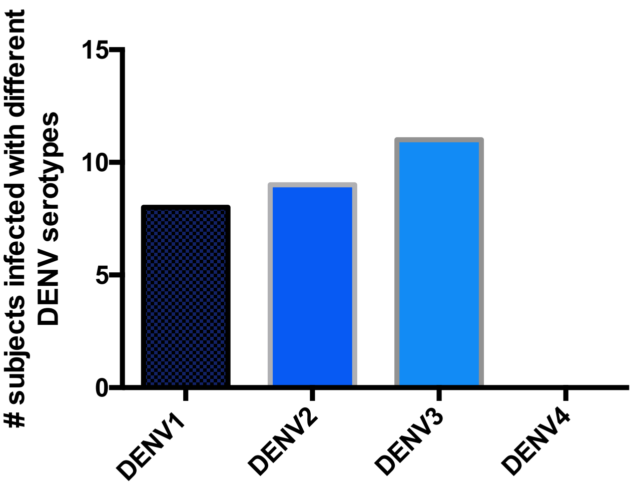

Supplementary Figure 8. Number of subjects from DENV-ZIKV and +DENV-ZIKV groups with prior DENV infections to the different DENV serotypes. Eight patients were previously infected with DENV1, 9 patients with DENV2, and 11 patients with DENV3.
