# Supplementary material for: Impact of pre-existing dengue immunity on human antibody and memory B cell responses to Zika
Source: Nat Commun. 2019 Feb 26;10:938. doi: 10.1038/s41467-019-08845-3 (PMC6391383; doi:10.1038/s41467-019-08845-3)
Supplement: Supplementary file 2 — Reporting Summary [file 41467_2019_8845_MOESM2_ESM.pdf]

nature research

Corresponding author(s): *Double-blind peer review submissions: write DBPR and your manuscript number here instead of author names.*

Reporting Summary

Nature Research wishes to improve the reproducibility of the work that we publish. This form provides structure for consistency and transparency in reporting. For further information on Nature Research policies, see Authors & Referees and the Editorial Policy Checklist.

Statistical parameters

When statistical analyses are reported, confirm that the following items are present in the relevant location (e.g. figure legend, table legend, main text, or Methods section).

n/a	Confirmed	
☐	☒	The exact sample size (n) for each experimental group/condition, given as a discrete number and unit of measurement
☐	☒	An indication of whether measurements were taken from distinct samples or whether the same sample was measured repeatedly
☐	☒	The statistical test(s) used AND whether they are one- or two-sided<br>*Only common tests should be described solely by name; describe more complex techniques in the Methods section.*
☒	☐	A description of all covariates tested
☒	☐	A description of any assumptions or corrections, such as tests of normality and adjustment for multiple comparisons
☐	☒	A full description of the statistics including central tendency (e.g. means) or other basic estimates (e.g. regression coefficient) AND variation (e.g. standard deviation) or associated estimates of uncertainty (e.g. confidence intervals)
☐	☒	For null hypothesis testing, the test statistic (e.g. F, t, r) with confidence intervals, effect sizes, degrees of freedom and P value noted<br>*Give P values as exact values whenever suitable.*
☒	☐	For Bayesian analysis, information on the choice of priors and Markov chain Monte Carlo settings
☒	☐	For hierarchical and complex designs, identification of the appropriate level for tests and full reporting of outcomes
☒	☐	Estimates of effect sizes (e.g. Cohen's d, Pearson's r), indicating how they were calculated
☐	☒	Clearly defined error bars<br>*State explicitly what error bars represent (e.g. SD, SE, CI)*

Our web collection on statistics for biologists may be useful.

Software and code

Policy information about availability of computer code

Data collection	No software was used for the collection of data.
Data analysis	Data analysis was performed with Graphpad Prism version 6 for Mac.

For manuscripts utilizing custom algorithms or software that are central to the research but not yet described in published literature, software must be made available to editors/reviewers upon request. We strongly encourage code deposition in a community repository (e.g. GitHub). See the Nature Research guidelines for submitting code & software for further information.

Data

Policy information about availability of data

All manuscripts must include a data availability statement. This statement should provide the following information, where applicable:

- Accession codes, unique identifiers, or web links for publicly available datasets
- A list of figures that have associated raw data
- A description of any restrictions on data availability

All main data supporting the findings are available within the article or the Supplementary Information. The other data are available from the authors upon reasonable request.

Field-specific reporting

Please select the best fit for your research. If you are not sure, read the appropriate sections before making your selection.

- [x] Life sciences
- [ ] Behavioural & social sciences
- [ ] Ecological, evolutionary & environmental sciences

For a reference copy of the document with all sections, see nature.com/authors/policies/ReportingSummary-flat.pdf

Life sciences study design

All studies must disclose on these points even when the disclosure is negative.

Sample size	Blood samples were collected between July 2016 and March 2017 in the community-based prospective Pediatric Dengue Cohort Study (PDCS) in Managua, Nicaragua from suspected Zika cases who presented with rash and with one or more of the following symptoms: conjunctivitis, arthralgia, myalgia, and/or peri-articular edema, regardless of fever. ZIKV infection was confirmed by real-time RT-PCR performed at the National Virology Laboratory of the Ministry of Health in Managua using either one of two triplex assays that simultaneously detect ZIKV, CHIKV and DENV infections: the ZCD assay or the CDC Trioplex assay. Samples from ZIKV-positive patients were collected at early (~14 days post-infection) and late (~8 months post-infection) convalescence. Peripheral blood mononuclear cells (PBMCs) from ZIKV-infected patients who were DENV-naïve (n=11) or who had previously been exposed to DENV once (n=12) or more than once (n=8) were selected.<br>When developing this study, our primary objective was to compare differences between Zika patients with no prior DENV exposure versus ZIKV-infected patients with previous DENV exposure. According to our statistical analysis, a minimum of 8 samples per group would allow us to draw confident conclusions between these groups. When available we included more samples per group.
Data exclusions	No data was excluded from the analysis.
Replication	Replication of the Multi-Color Fluorospot analysis was successful and data analyzed was consistent and reproducible.
Randomization	Randomization was not relevant to our study because we were not applying a treatment nor doing a case-control study.
Blinding	Blinding was not relevant to our study because we were not applying a treatment nor a control study.

Reporting for specific materials, systems and methods

Materials & experimental systems

n/a	Involved in the study
☒	☐ Unique biological materials
☐	☒ Antibodies
☒	☐ Eukaryotic cell lines
☒	☐ Palaeontology
☒	☐ Animals and other organisms
☐	☒ Human research participants

Methods

n/a	Involved in the study
☒	☐ ChIP-seq
☒	☐ Flow cytometry
☒	☐ MRI-based neuroimaging

Antibodies

Antibodies used	1. E95-mouse anti DENV1 antigen conjugated to Fluorophore Qdot-525. Obtained from M. Diamond (Washington University in St. Louis).<br>2. E96-mouse anti DENV2 antigen conjugated to Fluorophore Qdot-625. Obtained from M. Diamond (Washington University in St. Louis).<br>3. E88-mouse anti DENV4 antigen conjugated to Fluorophore Qdot-565. Obtained from M. Diamond (Washington University in St. Louis).<br>4. 5J7-human anti DENV3 antigen conjugated to Fluorophore Qdot-625. Obtained from J. Crowe (Vanderbilt University).<br>5. ZKA-64-mouse anti ZIKV antigen conjugated to Fluorophore Qdot-625. Obtained from D. Corti (HUMABS).<br>6. Goat anti-Human IgG (H+L) Secondary Antibody conjugated to Fluorophore TRITC. Supplier name Thermo Fisher Scientific. Catalog Number A18810. Lot number RRID AB_2535587.<br>7. 4G2- mouse anti Envelope flavivirus antigen conjugated to Fluorophore PE. Supplier Biomatik. Catalog#AB00230-2.0. Clone D1-4G2-4-15. Lot#T1619A11.
Validation	1. E95-anti DENV1 antigen . Primary murin antibody that reacts with DENV1 antigen. Validation: Shrestha, B., J. D. Brien, S. Sukupolvi-Petty, S. K. Austin, M. A. Edeling, T. Kim, K. M. O'Brien, C. A. Nelson, S. Johnson, D. H. Fremont, and M. S. Diamond. 2010. The development of therapeutic antibodies that neutralize homologous and heterologous genotypes of dengue virus type

1. PLoS Pathog. 6:e1000823.
2. E96-anti DENV2 antigen . Primary murin antibody that reacts with DENV2 antigen. Validation: Sukupolvi-Petty, S., S. K. Austin, M. Engle, J. D. Brien, K. A. Dowd, K. L. Williams, S. Johnson, R. Rico-Hesse, E. Harris, T. C. Pierson, et al. 2010. Structure and function analysis of therapeutic monoclonal antibodies against dengue virus type 2. J. Virol. 84: 9227–9239.
3. E88-anti DENV4 antigen . Primary murin antibody that reacts with DENV4 antigen. Validation: Sukupolvi-Petty, S., J. D. Brien, S. K. Austin, B. Shrestha, S. Swayne, K. Kahle, B. J. Doranz, S. Johnson, T. C. Pierson, D. H. Fremont, and M. S. Diamond. 2013. Functional analysis of antibodies against dengue virus type 4 reveals strain-dependent epitope exposure that impacts neutralization and protection. J. Virol. 87: 8826–8842.
4. 5J7-anti DENV3 antigen . Primary human antibody that reacts with DENV3 antigen. Validation: Fibriansah, G., J. L. Tan, S. A. Smith, R. de Alwis, T. S. Ng, V. A. Kostyuchenko, R. S. Jadi, P. Kukkaro, A. M. de Silva, J. E. Crowe, and S. M. Lok. 2015. A highly potent human antibody neutralizes dengue virus serotype 3 by binding across three surface proteins. Nat. Commun. 6: 6341.
5. ZKA-64-anti ZIKV antigen . Primary human antibody that reacts with ZIKV antigen. Validation: Stettler, K., Beltramello, M., Espinosa, D.A., Graham, V., Cassotta, A., Bianchi, S., Vanzetta, F., Minola, A., Jaconi, S., Mele, F. and Foglierini, M., 2016. Specificity, cross-reactivity and function of antibodies elicited by Zika virus infection. Science, p.aaf8505.
6. Antigen human IgG. Goat anti-Human IgG (H+L) Secondary Antibody. Validation: See manufacture's website.
7. 4G2-anti E-flavivirus antigen. Primary murin antibody that reacts with E antigen from Zika and dengue. Validation: Lambeth, C. R., White, L. J., Johnston, R. E., & De Silva, A. M. (2005). Flow cytometry-based assay for titrating dengue virus. Journal of clinical microbiology, 43(7), 3267-3272.

Human research participants

Policy information about studies involving human research participants

Population characteristics

The study involved 31 pediatric patients that were diagnosed with Zika infection who were either DENV-naïve (n=11) or who had previously been exposed to DENV once (n=12) or more than once (n=8) were selected. From the 31 patients, 12 were female and 19 were male. The mean age of the patients was 10 years.

Recruitment

Blood samples from suspected Zika cases who presented with rash and with one or more of the following symptoms: conjunctivitis, arthralgia, myalgia, and/or peri-articular edema, regardless of fever were collected between July 2016 and March 2017 in the community-based prospective Pediatric Dengue Cohort Study (PDCS) in Managua, Nicaragua. The Pediatric Dengue Cohort Study (PDCS) was reviewed and approved by the Institutional Review Boards (IRBs) of the University of California, Berkeley, USA and the Nicaraguan Ministry of Health. Parents or legal guardians of all subjects provided written informed consent, and subjects of 6 years of age and older provided oral assent. The protocol was amended in July 2015 to include screening for ZIKV infection in participants meeting the study case definition and again in February 2016 to expand the case definition. These amendments were approved by the IRBs reviewing the study.
